# Progressive, Sequential Non-arteritic Anterior Ischemic Optic Neuropathy in the Context of Metabolic Syndrome and a Crowded Disc: A Case Highlighting Synergistic Vascular Risk

**DOI:** 10.7759/cureus.98545

**Published:** 2025-12-05

**Authors:** Amirah Mohammad Razali, Muhammad Khairul Adha Fuaad, Muhammad Mohd Isa

**Affiliations:** 1 Department of Ophthalmology, Faculty of Medicine and Health Sciences, Universiti Putra Malaysia, Serdang, MYS; 2 Department of Ophthalmology, Hospital Sultan Abdul Aziz Shah, Universiti Putra Malaysia, Serdang, MYS

**Keywords:** anterior ischemic optic neuropathy (aion), non-arteritic, obstructive sleep apnoea, progressive, sequential

## Abstract

Non-arteritic anterior ischaemic optic neuropathy (NAION) is an ischaemic disorder involving the anterior portion of the optic nerve. It typically presents with acute, painless vision loss upon awakening, accompanied by optic disc oedema and an inferior altitudinal visual field defect. We report a 44-year-old woman with multiple metabolic risk factors, obstructive sleep apnoea, and an anatomically crowded optic disc, who developed progressive NAION followed by sequential involvement of the fellow eye, despite optimization of her risk factors.

## Introduction

Non-arteritic anterior ischaemic optic neuropathy (NAION) is one of the leading causes of irreversible vision loss in middle-aged and elderly individuals, with an incidence of 10-11 per 100000 [1,2]. NAION in individuals under the age of 50 is not rare, whereby Arnold et al. found that 108 (12.7%) of their cohort of patients were under 50 years with higher rates of eye involvement than in those 50 years and older [3]. Clinically, patients typically present with a sudden onset, painless, unilateral blurring of vision, a relative afferent pupillary defect, variable visual field defect, a hyperaemic swollen optic disc and the presence of one or more splinter haemorrhages [4]. The pathogenesis of this condition involves a few features, commonly involving infarction in the laminar and retrolaminar regions with flow impairment to the prelaminar optic disc during the acute phase and a small, crowded disc as a predisposing factor [5]. Multiple vascular risk factors have been identified, including diabetes and hypertension, on top of other systemic risk factors such as obstructive sleep apnoea and migraine [5-7]. Arnold et al. found that chronic renal failure and migraine were more common in those younger than 50 years old [3]. In most cases, the visual acuity will remain stable with 42% showing improvement in vision without intervention based on the Ischemic Optic Neuropathy Decompression Trial [8]. A small proportion of patients suffer from the progressive form defined by worsening visual acuity of at least three Snellen lines occurring within the first 30 days after initial visual loss [3]. Sequential NAION on the other hand is said to occur when the patient experiences fellow eye involvement in subsequent years. This occurs in 14.7% of patients over a period of five years [9]. We report a distinctive case of progressive non-arteritic anterior ischaemic optic neuropathy with subsequent sequential involvement, emphasizing its unique clinical course and delineating the critical learning points for diagnostic evaluation, risk assessment, and management strategies.

## Case presentation

A 44-year-old lady with underlying poorly controlled hypertension, diabetes mellitus, dyslipidaemia, morbid obesity and obstructive sleep apnoea, presented with a one-week history of sudden onset blurred vision affecting the lower half of her right visual field, noted shortly after waking up. She denied eye pain or pain on eye movement.

**Figure 1:**
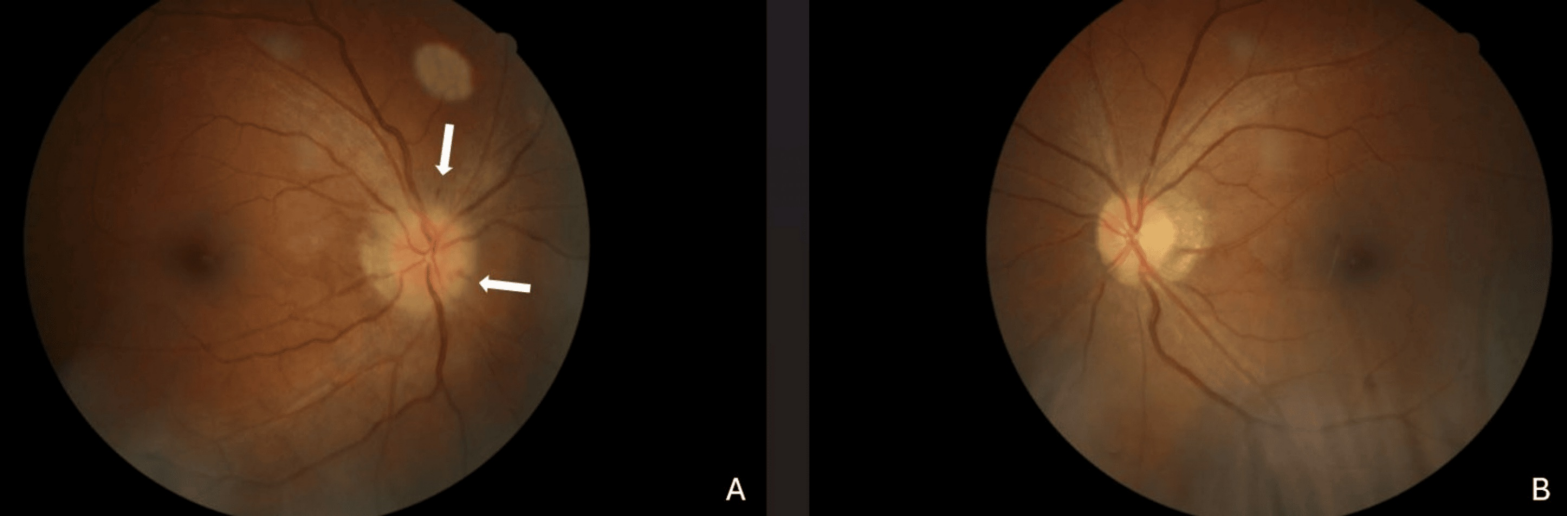
Fundus photo of the right eye (A) and the left eye (B) There is right optic disc swelling with splinter haemorrhages at 12 o’clock and 4 o'clock (white arrows), while the left eye showed a crowded optic disc.

On examination, visual acuity was 6/7.5 in the right eye and 6/6 in the left eye. A relative afferent pupillary defect was present in the right eye, with reduced light brightness. The anterior segment examination was normal with an intraocular pressure of 12mmHg bilaterally. Fundus examination revealed a swollen right optic disc with splinter haemorrhages at 12 and 4 o’clock, and a crowded disc in the left eye (Figure 1). No diabetic retinopathy was seen.

**Figure 2:**
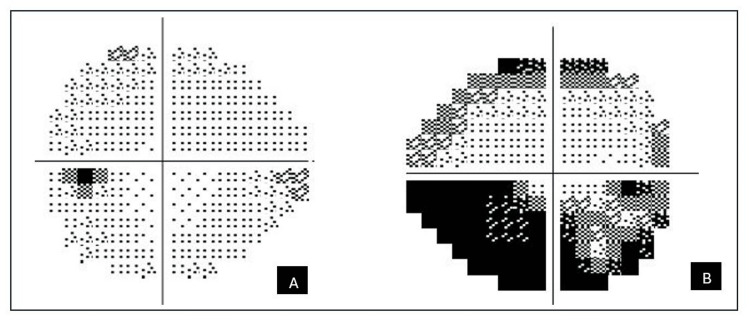
Humphrey visual field of the left eye (A) and the right eye (B). The Humphrey visual field is normal over the left eye whilst the right eye showed an inferior altitudinal visual field defect.

**Figure 3:**
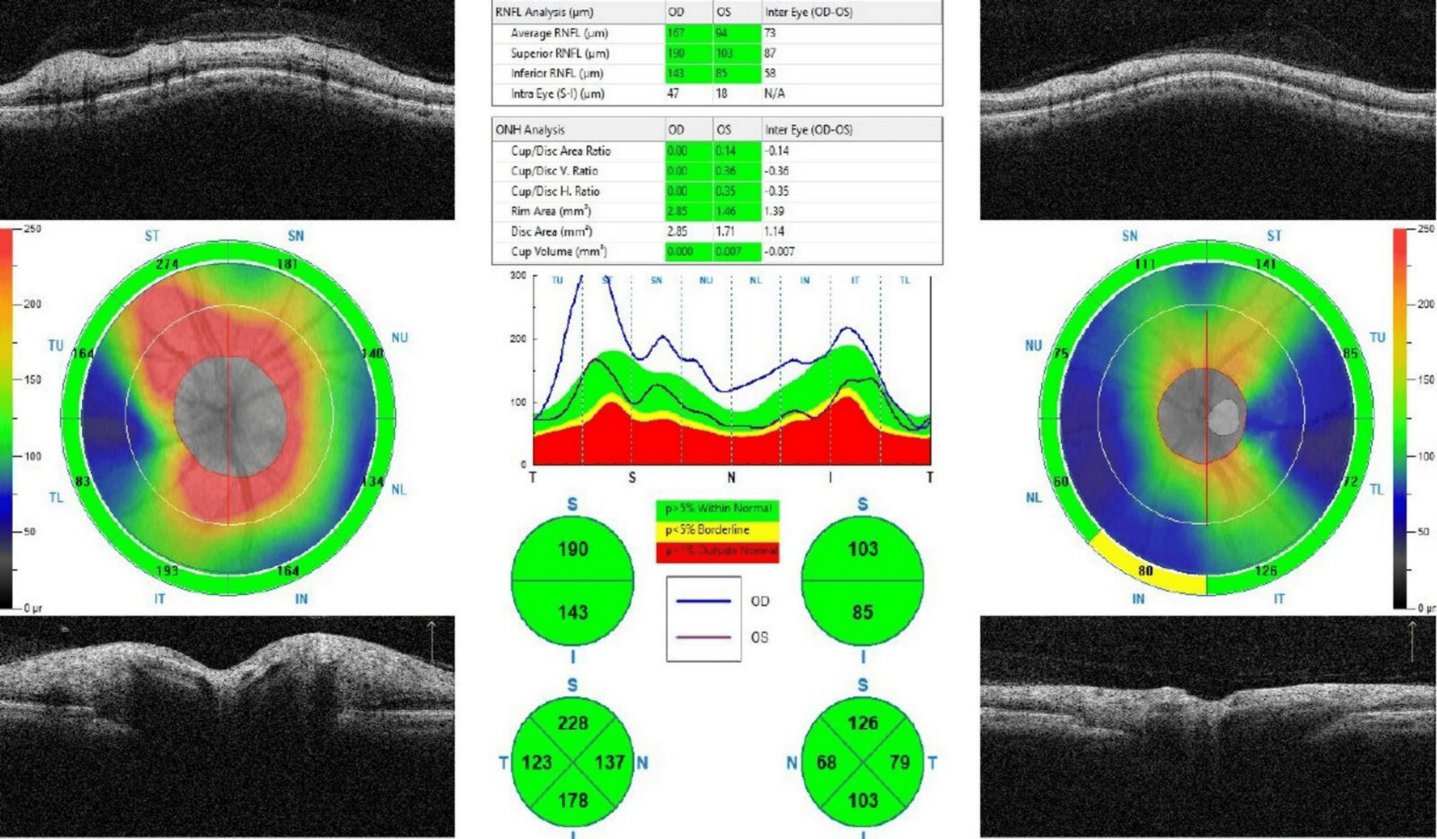
OCT of the retinal nerve fibre layer Optical coherence tomography (OCT) showing thickened peripapillary retinal nerve fibre layer of the right eye and normal thickness over the left eye

Systemically, her blood pressure was 180/110 mmHg with a random blood glucose of 11.0 mmol/L. Other systemic and neurological examination was unremarkable. Humphrey visual field test demonstrated a right inferior altitudinal visual field defect (Figure 2). Optical coherence tomography (OCT) showed diffuse thickening of the right peripapillary retinal nerve fibre layer as shown in Figure 3.

**Figure 4:**
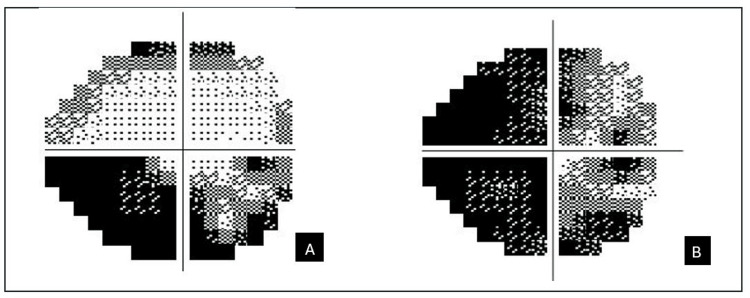
Humphrey visual field of the right eye at presentation (A) and at one month follow-up (B) At presentation, there was an inferior altitudinal visual field defect, which progressed to a generalized visual field defect, more marked nasally.

At one-month review, the visual field defect had progressed to involve the superior field within a week after her initial visit, but has remained stable thereafter. Visual acuity declined to 6/36, with a near-total visual field defect (Figure 4).

Blood investigations revealed poor metabolic control as shown in Table 1, with elevated fasting blood glucose level and abnormal lipid profile. Full blood count, renal and liver profiles were normal. Inflammatory markers were within normal limits with negative autoimmune screening as shown in Table 1. 

**Table 1:** Blood investigations together with reference range

Parameter	Result	Reference range
Haemoglobin	12.3 g/dL	12.0-15.0
White blood cell	8.67 x 10^3^/µL	4.0-10.0
Platelet	258 x 10^3^/µL	150.0-410.0
Fasting blood glucose	9.8 mmol/L	3.9-6.0
HbA1c	7.0 %	<5.7%
Total cholesterol	4.9 mmol/L	<5.2
Triglycerides	1.5 mmol/L	<1.7
Low-density lipoprotein	3.3 mmol/L	Low CV risk: <3.0 Intermediate CV risk: <3.8 High CV risk: ≤2.6 Very High CV risk: ≤2.2
High-density lipoprotein	0.9 mmol/L	0-1.2
C-reactive protein	10mg/L	1-5
Erythrocyte sedimentation rate	26mm/hr	
Anti-nuclear antibody	Negative	
Anti-double-stranded deoxyribonucleic acid antibody	<10 IU/mL	<100
C3	161 mg/dL	90-180
C4	36 mg/dL	10-40
Rheumatoid factor	Negative	

Contrast-enhanced computed tomography of the brain and orbit was unremarkable. She was referred for systemic optimisation, commenced on aspirin 100mg daily and underwent bariatric surgery. She was also initiated on continuous positive airway pressure (CPAP) therapy for her obstructive sleep apnoea and remained compliant.

**Figure 5:**
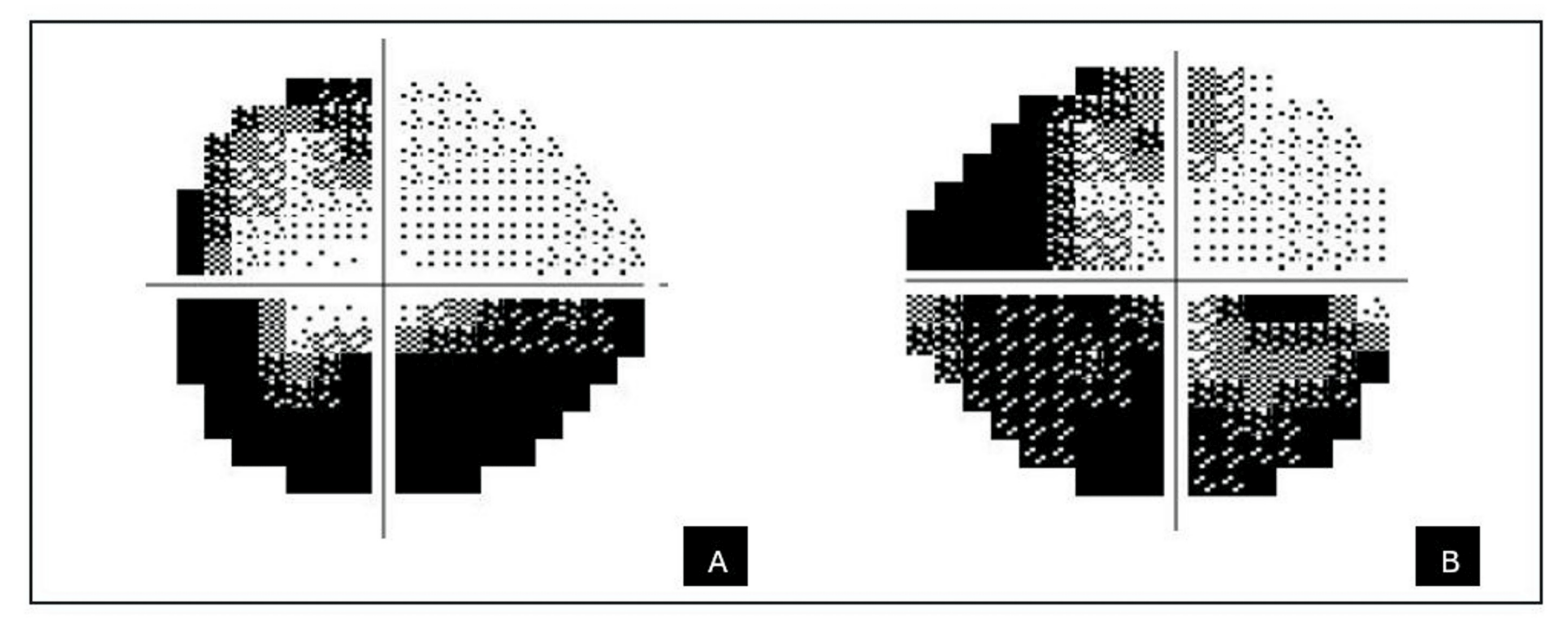
Humphrey visual field of the left eye (A) and the right eye (B) at 15 months follow-up The Humphery visual field showing a bilateral inferior altitudinal visual field defect

She remained stable until 15 months later, when she presented with similar symptoms in the left eye, describing blurring predominantly in the inferior field. Visual acuity was 6/12 in the right eye and 6/6 in the left eye. Anterior segment remained normal. The right optic disc appeared pale, while the left was swollen. Humphrey visual field testing showed bilateral inferior altitudinal visual field defects (Figure 5). 

**Figure 6:**
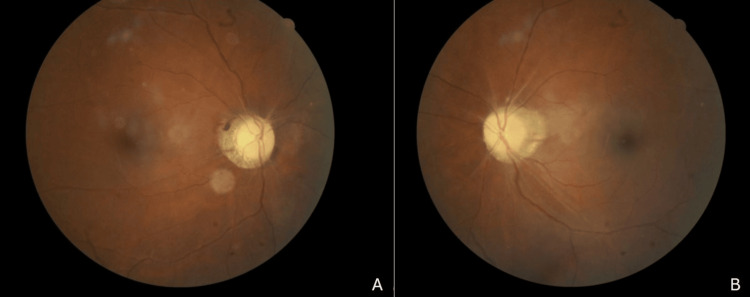
Fundus photo of the right eye (A) and the left eye (B) Fundus photo showing bilateral pale optic disc.

Repeated blood investigations demonstrated improved metabolic control with fasting glucose of 4.5 mmol/L, HbA1c of 4.8%, cholesterol level of 4.2 mmol/L, low-density lipoprotein level of 2.6 mmol/L and normal inflammatory markers. Her blood pressure ranged between 109-141/80-88 mmHg during clinic visits, and her BMI decreased from 53 to 41 kg/m2. Contrast-enhanced computed tomography of the brain and orbit was again unremarkable. A final diagnosis of sequential NAION was made. At six months follow-up, her best corrected visual acuity was 6/12 over the right eye and 6/6 over the left eye with bilateral optic disc pallor (Figure 6) and an inferior altitudinal visual field defect.

## Discussion

NAION is the commonest acute optic neuropathy in those over 50 years of age, which can lead to significant visual impairment [4]. The pathogenesis remains unclear with regard to cause of the ischaemic event, be it nocturnal hypotension, local atherosclerosis, vasospasm, impaired autoregulation, structurally small ‘crowded’ optic disc or a combination of these factors [4,5]. Hayreh categorizes the risk factors into two categories, which are predisposing factors and precipitating factors [6]. Predisposing factors may be systemic or ocular; systemic risk factors include diabetes, hypertension, hypotension, blood loss, cardiovascular disease and migraine, while local risk factors include poor optic nerve head blood supply, posterior ciliary artery disease, autoregulation impairment of the optic nerve head blood flow and raised intraocular pressure [6]. The main precipitating factor, according to Heyrah, is the nocturnal arterial hypotension leading to the transient hypoperfusion of the optic nerve head, occurring predominantly during sleep [6]. Other risk factors that have been discussed are obstructive sleep apnoea, migraine and medications such as antihypertensives, including beta-blockers and phosphodiesterase inhibitors [5,7,10]. In obstructive sleep apnoea, evidence suggests that optic nerve head damage occurs during the hypoxic events coupled with the increased intracranial pressure during apnoeic episodes which further impairs perfusion to the optic nerve head [7]. There is also an additional impaired autoregulation occurring during the event as a result of vasoactive substances imbalance [7].

Sequential NAION is uncommon, occurring in 14.7% of patients over a period of five years [9]. Based on a recent retrospective case control study of 76 patients, the risk factor profiles and visual outcomes of patients with simultaneous NAION seem to be similar to those with unilateral NAION, suggesting a similar pathophysiologic mechanism with the same risk factors [10]. They also found that in their cohort of patients, 10 patients developed incipient NAION, of which nine became symptomatic, leading to visual loss [10]. Karen et al. conducted a systematic review and meta-analysis on visual outcome following bilateral NAION and they found that the severity of the visual loss in the first eye cannot be used to predict the visual outcome the of sequential contralateral eye, and that actions taken after the initial NAION does not help in improving the visual outcome of the contralateral eye in the event it does occur [11]. This likely reflects a combination of factors, including bilateral similarity in optic disc anatomy and shared vasculopathic risk exposures. Our patient developed sequential NAION over a period of 15 months on a background of a small, crowded disc and multiple systemic risk factors. She initially had poorly controlled metabolic disease with hypertension, diabetes, hyperlipidaemia and obesity. Her blood pressure control was variable with multiple fluctuations. Despite attempts at optimizing her medical conditions, which include adjustment of her blood pressure and diabetic medications, bariatric surgery and commencement on a CPAP machine, she still developed NAION in the contralateral eye, but a less severe form. Her presenting blood pressure ranged from 128-180/94-101 mmHg, necessitating four antihypertensive medications to stabilise her blood pressure to 109-141/80-88 mmHg. Such intensive blood pressure reduction may have contributed to nocturnal arterial hypotension, resulting in transient optic nerve head hypoperfusion. In terms of visual outcome, her visual acuity was 6/12 in the right eye and 6/6 in the left eye, with bilateral inferior altitudinal visual field defects.

The natural history of NAION can be observed based on two large studies. A multicentre clinical trial by the Ischemic Optic Neuropathy Decompression Trial, 42% of patients had spontaneous improvement in visual acuity without any intervention, occurring highest at three months. There was also subsequent gradual reduction in vision over 24 months with final vision better than during presentation [12]. Heyreh et al. found that those with visual acuity of 20/70 and worse, 41% improved at six months and 42% at one year, but at two years, 9% of eyes had a drop in vision to 20/60 or worse. In terms of visual field defect, 43% of the 386 eyes had moderate to severe visual field defect, and in this cohort of patients, 26% had improvement at six months, 27% at one year and 19% had worsening visual field defects at two years [13]. Initial deterioration in vision seen in proportion of patients, grouped as the progressive type whereby there is progressive worsening of the vision over a few weeks after presentation before stabilizing. This usually occurs within two to three months, and if occurring beyond that, the patients should be assessed for other causes of optic neuropathy [4]. Progressive NAION occurs in 22% to 44% of patients, more frequently in younger patients [3]. No study has specifically evaluated progressive NAION and the risk of sequential NAION. In a retrospective review of 122 eyes, progressive and stable NAION shared similar demographics, systemic risk factor and initial visual deficient; the only distinguishing feature was earlier presentation in the progressive group [14]. Acute ischaemia induces axonal oedema and compartment compression particularly at the crowded region of the cribriform plate, resulting in secondary axoplasmic stasis, further axonal degeneration, impaired retrograde neurotrophin transport, and consequent retinal ganglion cell loss [5,15].

Management of NAION predominantly involves thorough evaluation and reduction of the risk factor for a particular patient. There is yet a consistent, effective therapy in treating NAION. Based on Ischaemic Optic Neuropathy Decompression Trial Research Group, optic nerve sheath decompression was found to not be effective and may be harmful as 24% of eyes further developed visual loss as opposed to only 12% that had no treatment [8]. High-dose systemic steroid therapy has been used, though it remains controversial. The optic disc swelling induces a compartment syndrome whereby axons are compressed within a small scleral opening, and giving steroids helps to expedite the resolution of the oedema, relieving compression and enhancing blood flow to the ischaemic axons [16]. Although it helps hasten the process, there seem to be no significant differences in the final visual outcome for the patients. Zhao et al. grouped patients into four groups, group one had no treatment, group two received oral prednisolone 1mg/kg/d for 14 days, group three received 250 units methylprednisolone once daily for three days followed by oral prednisolone 1mg/kg/d for 11 days, and group four received 500mg units methylprednisolone once daily for three days followed by oral prednisolone 1mg/kg/d for 11 days [16]. There was no significant difference in final visual outcome, but it was suggested that 500 units of methylprednisolone resulted in improvement in short-term visual acuity and may be considered for patients needing to enhance the short-term vision with no contraindications to high-dose steroid therapy [16]. The use of aspirin has also been discussed. There seems to be no long-term benefit of using aspirin to reduce the risk of simultaneous NAION, nor does it improve visual outcome, as NAION is not a thromboembolic disorder but a hypotensive disorder [17]. A recent systematic review and meta-analysis by Lantos et al. looking at efficacy of treatments in NAION which included therapies such as steroids, oxygen, steroids plus erythropoietin, levodopa/carbidopa, memantine, anticoagulants and thrombolytics, and they concluded that there are no effective treatment for NAION, but by eliminating cardiovascular risk factors, the incidence of NAION can be reduced [18].

## Conclusions

Early identification and optimization of vascular and systemic factors remain the key to preventing bilateral NAION. Multidisciplinary management involving ophthalmologists, physicians and sleep specialists, together with patient education, is crucial to reduce the overall risk and prevent permanent visual disability. Regular systemic monitoring, careful medication adjustments and awareness of nocturnal hypotension are vital in reducing the risk of further ischemic events.
